# Optoelectronic frequency-modulated continuous-wave terahertz spectroscopy with 4 THz bandwidth

**DOI:** 10.1038/s41467-021-21260-x

**Published:** 2021-02-16

**Authors:** Lars Liebermeister, Simon Nellen, Robert B. Kohlhaas, Sebastian Lauck, Milan Deumer, Steffen Breuer, Martin Schell, Björn Globisch

**Affiliations:** 1 Fraunhofer Institute for Telecommunications, Heinrich Hertz Institute, Berlin, Germany; 2 Institut für Festkörperphysik, Technische Universität Berlin, Berlin, Germany

**Keywords:** Optoelectronic devices and components, Terahertz optics, Optical spectroscopy, Terahertz optics, Optical spectroscopy

## Abstract

Broadband terahertz spectroscopy enables many promising applications in science and industry alike. However, the complexity of existing terahertz systems has as yet prevented the breakthrough of this technology. In particular, established terahertz time-domain spectroscopy (TDS) schemes rely on complex femtosecond lasers and optical delay lines. Here, we present a method for optoelectronic, frequency-modulated continuous-wave (FMCW) terahertz sensing, which is a powerful tool for broadband spectroscopy and industrial non-destructive testing. In our method, a frequency-swept optical beat signal generates the terahertz field, which is then coherently detected by photomixing, employing a time-delayed copy of the same beat signal. Consequently, the receiver current is inherently phase-modulated without additional modulator. Owing to this technique, our broadband terahertz spectrometer performs (200 Hz measurement rate, or 4 THz bandwidth and 117 dB peak dynamic range with averaging) comparably to state-of-the-art terahertz-TDS systems, yet with significantly reduced complexity. Thickness measurements of multilayer dielectric samples with layer-thicknesses down to 23 µm show its potential for real-world applications. Within only 0.2 s measurement time, an uncertainty of less than 2 % is achieved, the highest accuracy reported with continuous-wave terahertz spectroscopy. Hence, the optoelectronic FMCW approach paves the way towards broadband and compact terahertz spectrometers that combine fiber optics and photonic integration technologies.

## Abstract (web summary)

Time-domain spectroscopy with terahertz frequencies typically requires complex and bulky systems. Here, the authors present an opto-electronics-based, frequency-domain terahertz sensing technique which offers competitive measurement performance in a much simpler system.

## Introduction

The potential of terahertz (THz) spectroscopy for applications in fundamental science and industrial non-destructive testing (NDT) has long been recognized^1,2^. In scientific applications, intensive THz pulses can control the electronic, ionic, and spin degrees of freedom in matter^3^. In NDT, broadband terahertz spectroscopy has been successfully explored for non-contact thickness measurements of multilayer dielectric coatings^4–7^ and sensing of defects in polymers, foams, and other non-conductive materials^8–10^. Today, most of the aforementioned applications make use of THz TDS systems, which convert the enormous bandwidth of an optical femtosecond pulse into the THz domain, using either an ultrafast photoconductive switch or a nonlinear crystal^11–14^. In the last decade, compact femtosecond lasers and the use of devices and components, which were originally developed for fiber-based telecommunications, enabled the development of table-top THz TDS systems, the best of which now attain a bandwidth of 6.5 THz and peak dynamic range (DR) of more than 100 dB^15–17^. However, the fundamental drawback of these systems is their high complexity. They require expensive femtosecond pulsed laser sources and an elaborate optical delay based either on free-space optics and optomechanics^16^, or cavity detuning^18^, or the complex synchronization^19^ of two laser sources^7,20,21^. Consequently, all three approaches make system assembly and adjustment demanding, or require complex electronic control schemes. Hence, simpler approaches for generating and detecting broadband THz signals are highly desirable.

Optoelectronic continuous-wave THz (cw THz) spectroscopy^22,23^, on the other hand, is a promising technique for applications that require high frequency resolution rather than high measurement rates^24^. However, cw THz technology has the potential to address applications in other areas of sensing, industrial NDT, and imaging as well: First, the all-fiber design of cw THz systems removes the need of any free-space optics, moving parts, or mechanical delay lines. Second, the use of cw lasers instead of complex femtosecond pulse lasers presents a key advantage, as cw lasers are semiconductor chips that can be mass-produced at low costs. For a long time, the main limitation of cw THz systems seemed to be their acquisition rate of a few measurements per minute at best. In a very recent publication, we presented an all-fiber-based cw THz system with 2 THz bandwidth at a measurement rate of 24 Hz^25^. To this end, our previous work combined a rapidly swept cw laser with an optical phase modulator for broadband and phase-sensitive measurements.

In this work, we present a fast and coherent cw terahertz spectrometer that uses a swept delayed self-heterodyning (S-DSH) technique, originally developed to measure the linewidth of lasers^26^. The spectrometer includes a swept laser source, a fixed-frequency cw laser, an erbium-doped fiber amplifier (EDFA), two photomixers for THz generation and detection, and electronics for data acquisition and processing. Owing to the S-DSH technique, an active phase or amplitude modulation—the standard technique for coherent cw THz measurements so far—is no longer required. This technique can be regarded as an optoelectronic analog of frequency-modulated continuous-wave (FMCW) radar^27,28^. Our THz spectrometer achieves a bandwidth of 4 THz, a peak DR of 117 dB, and a measurement speed of 200 Hz, i.e., a performance comparable to that of state-of-the-art THz TDS systems, yet without their complexity. With this spectrometer, we demonstrate broadband spectroscopy and highly accurate NDT, such as contract-free multilayer thickness measurements.

## Results

In this study, we apply a swept S-DSH system to photomixing-based coherent frequency-domain THz spectroscopy. The S-DSH technique has long been used to analyze the temporal fluctuations of a laser source^29,30^. We show that the same technique lends itself to broadband THz measurements as well as fast thickness gauging.

This section starts with a detailed description of our optoelectronic implementation of the S-DSH technique. Afterwards, we analyze the influence of the frequency tuning scheme and the coherence properties of the swept laser source. Finally, we characterize the coherent THz S-DSH spectrometer with respect to bandwidth and DR.

### THz FMCW with S-DSH

**Fig. 1:**
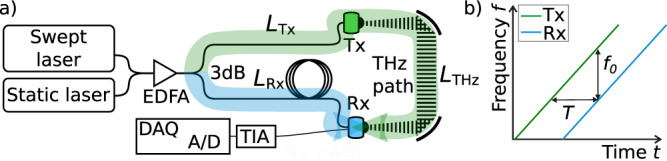
Schematic setup and principle of operation of the optoelectronic FMCW spectrometer. **a** Schematic drawing of the optoelectronic swept delayed self-heterodyning FMCW THz setup. Note that the system is fully fiber-coupled without any free-space optics. EDFA erbium-doped fiber amplifier, 3 dB 3 dB coupler, *L*_Tx_, *L*_R*x*_ optical fiber path length in the Tx and Rx arm, respectively, including an optional static fiber delay; *L*_THz_ THz path length, Tx fiber-coupled THz emitter, Rx fiber-coupled THz receiver, TIA transimpedance amplifier, DAQ data digitization and acquisition system. The colored arrows highlight the Tx path and Rx path, and indicate the path lengths relevant for the total delay time that arises from the path length asymmetry. **b** When the frequency *f* of the swept laser is tuned at constant velocity *ν*_S_, a frequency offset *f*_0_ = *T* d*f*/d*t* occurs for a delay time *T*.

The system architecture of our optoelectronic FMCW THz spectrometer resembles a standard photomixing-based cw THz setup^23^. A schematic drawing is shown in Fig. 1. An optical beat note at the desired THz frequency is generated by superimposing a fixed-frequency laser (static laser) with the swept-frequency laser in a first 3 dB coupler. Both lasers are fiber-coupled and operate in the C-band. An EDFA boosts the power of the beat note before the output is split into two arms of equal power by a second 3 dB coupler. One output illuminates the photomixer emitter, which converts the optical beat to THz radiation. A pair of parabolic mirrors guide the THz signal from the emitter to the receiver. In the receiver, the THz signal is mixed with the optical beat note of the second optical arm. This mixing process generates a receiver current, which is digitized and processed to obtain the measurement result. Note that this system is entirely fiber-coupled and does not contain any free-space optics or moving parts. Further technical details are provided in the “Methods” section.

The conceptual novelty of our approach is the utilization of S-DSH for phase-sensitive detection. The main idea is the following: At the receiver, the THz field is mixed with an optical beat note, which is a time-delayed copy of the beat note that generated the THz field. As the beat note is swept in frequency, this delayed self-heterodyning results in a frequency difference between the THz field and the optical beat note at the receiver (see Fig. 1b). Due to the mixing process, the receiver current oscillates at an intermediate frequency, while preserving the amplitude and phase information of the THz field. Therefore, the intermediate frequency can be used to recover amplitude and phase of the THz signal. In the following, this scheme is derived in detail.

The complex detector current *J*(*t*) in the receiver results from photomixing of the incoming THz electric field *E*_THz,Rx_(*t*) and the intensity of the optical beat note *I*_B,Rx_(*t*)

1 $$J\left( t \right) = E_{{\mathrm{{THz,Rx}}}}\left( t \right)I_{{\mathrm{{B,Rx}}}}\left( t \right) = \left| {E_{{\mathrm{{THz,Rx}}}}\left( t \right)} \right|\left| {I_{\mathrm{{B,Rx}}}\left( t \right)} \right|{\mathrm{e}}^{i\phi (t)} = J_0{\mathrm{e}}^{i\phi (t)}.$$

Since the detected signal is the real part of the complex current *J*(*t*), the signal amplitude *J*_0_ is only determined correctly if the phase *ϕ*(*t*) between the THz field *E*_THz,Rx_(*t*) and the optical beat note *I*_B,Rx_(*t*) is known. In general, the phase cannot be calculated a priori, since the optical path length, which mainly determines the phase, is not known with sufficient accuracy. Therefore, coherent optoelectronic THz spectrometers commonly require a phase modulation by at least 2*π*. In general, *ϕ*(*t*) can be expressed by the time delay *T* between *E*_THz,Rx_ (*t*) and *I*_B,Rx_(*t*), multiplied with the angular frequency of the optical beat note *ω*_B_

2 $$\phi \left( t \right) = T\omega _{\mathrm{B}}.$$

The total differential of the phase *ϕ*(*t*) reads

3 $$\frac{{{\mathrm{d}}\phi \left( t \right)}}{{{\mathrm{d}}t}} = \omega _{\mathrm{B}}\frac{{\partial T}}{{\partial t}} + T\frac{{\partial \omega _{\mathrm{B}}}}{{\partial t}}.$$

Equation (3) shows that phase modulation can be obtained in two different ways, either by varying the time delay *T* while the angular frequency of the beat note *ω*_B_ is kept constant, or vice versa. Most phase modulation techniques published thus far have taken advantage of the first term of Eq. (3), i.e. *T* is varied by a phase modulator^31^, a fiber stretcher^32^, or a free-space optical delay line^23^ while *ω*_B_ is kept constant. In contrast, our optoelectronic FMCW THz setup utilizes the second term of Eq. (3). The angular frequency of the beat note *ω*_B_ is swept while the time difference *T* remains non-zero but constant. Note that the variation of *ω*_B_ instead of *T* is particularly convenient in a broadband and coherent cw THz system, because fast frequency tuning of the beat note *ω*_B_ is required anyway for broad spectral coverage. Hence, akin to standard FMCW radar, our optoelectronic FMCW THz method exploits frequency sweeping not only for tuning but also for coherent detection.

The frequency *f* of the tunable laser, or equivalently, the beat note *ω*_B_, sweeps at a constant rate $\nu_{\mathrm{s}}$,

4 $$\nu _{\mathrm{S}} = 2\pi \frac{{{\mathrm{d}}f}}{{{\mathrm{d}}t}} = \frac{{{\mathrm{d}}\omega _{\mathrm{B}}}}{{{\mathrm{d}}t}} = {\mathrm{const}}.$$

With a constant time delay *T* between the incoming THz field and the optical beat note at the receiver (see Fig. 1b), the phase change in Eq. (3) then becomes

5 $$\frac{{{\mathrm{d}}\phi \left( t \right)}}{{{\mathrm{d}}t}} = T\nu _{\mathrm{S}} = 2\pi f_0$$

where *f*_0_ is the intermediate frequency. In our setup, the time delay *T* is given by the length of the optical fibers as well as the length of the THz path. To illustrate the effect, the Tx path (green) is drawn substantially longer than the Rx path (blue) in Fig. 1a. The time delay *T* can be expressed in terms of path lengths

6 $$T = \frac{{nL_{{\mathrm{Tx}}} + L_{{\mathrm{THz}}} - nL_{{\mathrm{Rx}}}}}{c}.$$

Here, *L*_Tx_ and *L*_Rx_ denote the fiber lengths in the emitter and receiver arm, respectively, *n* is the refractive index of the fiber and *L*_THz_ is the THz path length. Note that for a given THz path length *L*_THz_, the total time delay can be adjusted via the lengths of the static fibers *L*_Rx_ and *L*_Tx_, which either complement or compensate the THz path. This is most convenient, as the setup supports essentially any THz path length, without the need to modify the clock rate, filter bandwidths, or any electrical signaling such as the frequency tuning controls or the receiver current. According to Eqs. (5) and (6), the intermediate frequency *f*_0_ depends on the THz path length and therefore the introduction of a sample, or any other modification of this path, will shift the intermediate frequency.

The phase of the detector current is obtained by integrating Eq. (2) by *t* and inserting (5):

7 $$\phi \left( t \right) = 2\pi f_0t + \phi _0.$$

As expected, the phase rotates at the intermediate frequency *f*_0_ and includes a phase offset *ϕ*_0_, which is initially undefined. With a software quadrature lock-in running at a frequency close to *f*_0_ the phase offset can be calculated. Comparing the offset *ϕ*_0_ of a sample measurement with a reference measurement then yields an accurate measurement of the phase change introduced by the sample.

Combining Eqs. (5) and (1), we find for the detector current:

8 $$J\left( t \right) = J_0{\mathrm{e}}^{iT\nu _{\mathrm{S}}t} = \left| {E_{{\mathrm{THz,Rx}}}\left( t \right)} \right|\left| {I_{{\mathrm{B,Rx}}}\left( t \right)} \right|{\mathrm{e}}^{iT\nu _{\mathrm{S}}t/c + i\phi _0}.$$

This is the essential equation of the optoelectronic FMCW THz scheme. It shows that the receiver current is proportional to the THz field amplitude, and its phase is determined by the properties of the THz path. Therefore, both amplitude and phase information can be extracted from the receiver current.

In conclusion, the prerequisites for an optoelectronic FMCW THz system are a static asymmetry in path lengths, and a constant sweeping speed $\nu_{\mathrm{S}}$ of the optical beat note. If these requirements are fulfilled, the incident THz signal is mixed down to an intermediate frequency *f*_0_. With a software-based lock-in detector, amplitude and phase information of the THz signal are retrieved from the detector current. According to Eq. (5), the delay time *T* and the frequency tuning speed $\nu_{\mathrm{S}}$ of the swept laser source are the principal parameters that determine the intermediate frequency *f*_0_. For an optoelectronic FMCW spectrometer, the delay time *T* should be as small as possible in order to minimize any detrimental effects, which this additional delay may have on the phase and amplitude stability of the system. This means that $\nu_{\mathrm{S}}$ has to be sufficiently large. In the next two sections, we first derive an expression for the case of stepwise frequency tuning. We then discuss the interplay of coherence and the available parameter space for our optoelectronic FMCW spectrometer.

### Stepwise frequency tuning

According to Eq. (5), a constant intermediate frequency *f*_0_ requires a linear sweep of the laser frequency. However, the laser employed in this work operates with quasi-stepwise tuning. More precisely, the laser dwells at a certain frequency *f* for a time *τ*_s_ before it switches quasi instantaneously to the next frequency *f* + Δ*f*. Hence, the mean sweep rate is

9 $$\overline \nu _{\mathrm{S}} = \frac{{{\Delta}f}}{{\tau _{\mathrm{s}}}}.$$

**Fig. 2:**
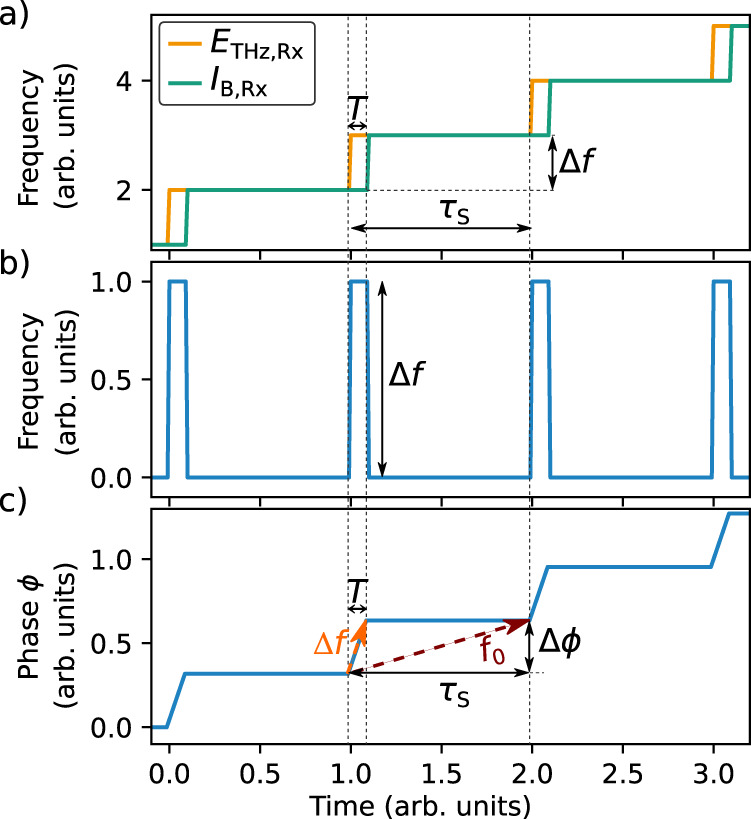
Illustration of the time-dependent detector signal of a FMCW THz spectrometer with stepwise frequency tuning. **a** Frequency of the incident THz field *E*_THz,Rx_ and the optical beat note *I*_B,Rx_ at the receiver as a function of time. The time delay *T* between *E*_THz,Rx_ and *I*_B,Rx_, the frequency step size Δ*f* and the cycle time *τ*_s_ are indicated. **b** Time-dependent frequency and **c** phase of the electrical signal after photomixing in the receiver. Frequency and phase of the receiver signal change by Δ*f* and Δ*ϕ*, respectively, within the time *T*. The quantity *f*_0_ denotes the linearized intermediate frequency, which is used for signal detection in our realization of the FMCW THz spectrometer.

At the receiver, the stepwise frequency tuning of the swept laser and the time delay between the Tx and Rx path causes the THz field *E*_THz,Rx_(*t*) to follow each step Δ*f* by the time *T* earlier than the optical beat note *I*_B,Rx_(*t*). This behavior is sketched in Fig. 2a. Note that the frequencies of the two signals *E*_THz,Rx_(*t*) and *I*_B,Rx_(*t*) are equal within each time interval *τ*_s_, except for the short period *T*. The detector current *J*(*t*) is modulated at the difference frequency of *E*_THz,Rx_(*t*) and *I*_B,Rx_(*t*), and the time course of this intermediate frequency thus follows a boxcar-shaped modulation between 0 and Δ*f* with a duty cycle of *T*/*τ*_s_, as shown in Fig. 2b. Therefore, the main difference between continuous and stepwise frequency tuning is the following: In the former case, the intermediate frequency is constant and accurately obeys Eq. (5). In the case of stepwise tuning, however, the intermediate frequency remains zero within the time period *τ*_s_ except for the short time interval *T*. The phase *ϕ*(*t*) of the detector current then follows the sequence shown in Fig. 2c. Note that the phase change occurs only within the short time interval *T* whereas the phase remains constant for the remainder of the time *τ*_s_. One might assume that stepwise frequency tuning requires much higher sampling speeds compared to continuous tuning, in order to resolve the phase change within the short time interval *T*. However, since the spectral resolution of the THz measurement equals the frequency step size Δ*f*, only the total phase Δ*ϕ* acquired within one frequency step matters. The detailed time course of the phase within *τ*_s_ can thus be neglected. Hence, the assumption of a linear phase evolution between two frequency steps suffices, as depicted by the dashed red line in Fig. 2c. In practice, this linearization is simply implemented by removing the higher harmonics of the intermediate frequency *f*_0_ in the detector current. This can be done, for example, with low-pass electronic filters prior to the digitalization of the receiver signal. The result is a constant intermediate frequency *f*_0_, which serves for phase-sensitive detection the same way as in the case of linear frequency tuning. From the graphical representation in Fig. 2c it can be derived that

10 $$f_0 = \frac{{{\Delta}\phi }}{{\tau _{\mathrm{s}}}} = {\Delta}f\frac{T}{{\tau _{\mathrm{s}}}}.$$

Hence, *f*_0_ is determined by the tuning frequency Δ*f* multiplied by the duty cycle *T*/*τ*_s_. Vice versa, Eq. (10) can be used to calculate the required time delay *T* for any desired intermediate frequency *f*_0_. In our experiments, the swept laser tunes in steps of Δ*f* = 1 GHz with a cycle time of *τ*_s_ = 2 µs. In order to accurately sample the phase within *τ*_s_, we choose *T* such that *f*_0_ amounts to approximately 500 kHz. In our optoelectronic FMCW experiment, the delay time *T* originates from an optical fiber (length 8.8 cm, refractive index *n* = 1.45) inserted between the 3 dB coupler and the Rx, which makes the Rx arm 12.8 cm longer than the Tx arm. The extra fiber also partially compensates the length of the THz path (*L*_THz_ = 40 cm) such that the total path length asymmetry between emitter and receiver becomes 27.2 cm, corresponding to a total delay time of *T* = 0.91 ns. Hence, Eq. (10) yields the intermediate frequency

11 $$f_0 = {\Delta}f\frac{T}{{\tau _{\mathrm{s}}}} = 1{\,}{\mathrm{GHz}}\frac{{0.91{\mathrm{ns}}}}{{2{\,}{\upmu {\mathrm{s}}}}} = 455{\,}{\mathrm{kHz}}.$$

**Fig. 3:**
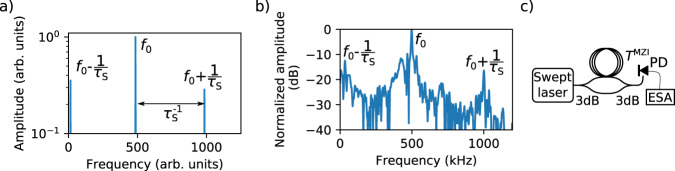
Calculated and measured spectra as acquired from the sweeping laser in an asymmetric MZI. **a** Spectrum of the detector current, as calculated from the amplitude and phase evolution shown in Fig. 2b, c. The center frequency *f*_0_ and the first higher-order components with frequencies *f*_0_ ± 1/*τ*_s_ are indicated. **b** Measured frequency spectrum of the photodetector in the asymmetric Mach–Zehnder interferometer experiment (see **c**) and Sec. 5.2. The graph shows the absolute value of the Fourier transform of 512,000 data points, recorded at a sampling rate of 80 MS/s. The time delay *T*^MZI^ = 1 ns was realized with a 20.6-cm-long optical fiber. Together with the tuning speed (500 THz/s) of the laser, the fundamental modulation frequency of the detector current is $f_0^{{\mathrm{{MZI}}}}$ = 498 kHz. Higher harmonics stem from the stepwise tuning scheme of the laser source and can be eliminated from the spectrum by a band pass filter centered at *f*_0_. **c** Setup of the Mach–Zehnder interferometer experiment, with a fiber delay *T*^MZI^, 3 dB couplers (3 dB), and a photodiode (PD) connected to an electrical spectrum analyzer (ESA).

The suitability of the sweeping laser and its stepwise frequency tuning for this detection scheme can be experimentally verified in a much simpler setup, without any THz components. We used a simple asymmetric Mach–Zehnder interferometer (MZI) with a standard photodetector^26^ on one output port to demonstrate the generation of *f*_0_ and its higher harmonics. This setup omits the conversion into THz radiation or the detection thereof, with the advantage that neither bandwidth limitations of THz generation and detection, nor any THz path adjustment, nor the limits of a high-gain current amplifier need to be considered. The setup is sketched in Fig. 3c and described in more detail in the “Methods” section. We note that the values for both the time delay *T* and the intermediate frequency of this setup differ slightly from those of the optoelectronic FMCW system. The values used in the interferometer are indicated by the superscript MZI. The detector current of the photodetector *J*^MZI^(*t*) is equivalent to *J*(*t*) in Eq. (1), if the THz field and optical beat note are replaced by the electric fields of the sweeping laser in the two arms *E*_1_(*t*) and *E*_2_(*t*). Using Eqs. (7) and (8), the photodetector current reads

12 $$J^{{\mathrm{MZI}}}\left( t \right) = E_1\left( t \right){\,}E_2\left( t \right) = J_0^{{\mathrm{MZI}}}{\mathrm{e}}^{i\phi (t)} = J_{\mathrm{0}}^{{\mathrm{MZI}}}{\mathrm{e}}^{iT^{{\mathrm{MZI}}}\nu _{\mathrm{S}}t}.$$

Obviously, the expression for the photodetector current in this setup closely resembles that of the THz detector current in the optoelectronic THz spectrometer. Therefore, the simple MZI setup allows us to conveniently analyze the properties of the laser, without taking any possible effects of the THz conversion into account. In our test, the interferometer features a fiber of 20.6 cm length, which gives rise to a time delay of *T*^MZI^ = 1 ns. This value is close, but not equal, to the delay *T* used in the optoelectronic FMCW THz setup. Figure 3a shows the frequency spectrum of the photodetector current, as obtained by Fourier transformation of Fig. 2b, c. For comparison, Fig. 3b depicts experimental data measured with the actual photodetector. Note that the frequency with the highest spectral amplitude corresponds to the linearized intermediate frequency $f_0^{{\mathrm{MZI}}} = 498$ kHz. The first harmonic of *f*_0_, a result of the stepwise frequency tuning of the swept laser source, is clearly observed at around 1 MHz. By applying a set of analog and digital band pass filters around the modulation frequency *f*_0_, both higher harmonics and noise are suppressed, while *f*_0_ remains available for phase-sensitive detection. It is worth noting that the filter bandwidth defines the scan depth of the THz spectrometer. In our realization, a filter bandwidth of 100 kHz corresponds to a scan depth of 6 cm, which is sufficient for the majority of applications, such as layer thickness measurements (see Discussion).

### Coherence

In optoelectronic cw THz systems, the coherence of both lasers translates directly into the coherence of the THz radiation^23,33^. For our setup, we assume that the temporal coherence of the swept laser source is much lower than the coherence time of the fixed-frequency laser. However, the common definition of temporal coherence, i.e. the DC component of the temporal interference, cannot be used to derive the coherence property of a swept laser, since this component vanishes by definition. Therefore, we use an approach adopted from ref. ^26^, which describes the sweeping angular frequency *ω*_s_(*t*) as the sum of an ideal linear sweep and a small deviation Δ*ω*_s_(*t*):

13 $$\omega _{\mathrm{S}}\left( t \right) = \omega _0 + \nu _{\mathrm{S}}t + {\Delta}\omega _{\mathrm{S}}\left( t \right).$$

Hence, the detected signal *Re*(*J*(*t*)) follows the difference frequency of the beat signal at times *t* and *t* + *T*. Consequently, the modulation frequency varies over time, due to imperfections in the sweep rate:

14 $$2\pi \left( {f_0 + {\Delta}f\left( t \right)} \right) = \omega _{\mathrm{S}}\left( t \right) - \omega _{\mathrm{S}}\left( {t + T} \right) = \nu _{\mathrm{S}}T + {\Delta}\omega _{\mathrm{S}}\left( t \right) - {\Delta}\omega _{\mathrm{S}}\left( {t + T} \right).$$

Taking this view, the linewidth Δ*f*_0_ of the intermediate frequency results from imperfections in the sweep at a time delay *T*:

15 $$2\pi {\Delta}f_0\left( t \right) = {\Delta}\omega_{\mathrm{S}}\left( t \right) - {\Delta}\omega_{\mathrm{S}}\left( {t + T} \right).$$

In order to ensure that the linewidth Δ*f*_0_(*t*) remains much smaller than the modulation frequency *f*_0_, i.e., Δ*f*_0_ ≪ *f*_0_, any deviations from the ideal sweep rate Δ*ω*_s_(*t*) have to be small on the time scale of *T*. Therefore, *T* must be less than both the coherence time of the static laser and the time scale of the fluctuations of the swept laser. The fast tuning speed of the swept laser source in the optoelectronic FMCW THz setup (see “Methods” section) enables us to choose a delay time *T* below 1 ns, which is much less than the coherence time of the static laser. As the linewidth of the intermediate frequency is dominated by the sweeping laser, it can be determined with the asymmetric MZI mentioned in the previous section. From Fig. 3, we infer a 3 dB linewidth of the modulation frequency *f*_0_ of Δ*f*_0_ = 5 kHz. This is less than 1% of the modulation frequency and hence the requirement Δ*f*_0_ ≪ *f*_0_ is met. We conclude that a swept laser source with quasi-stepwise tuning, with a speed of 500 THz/s, is well suited to apply the S-DSH concept to coherent terahertz spectroscopy

### Bandwidth and DR of the FMCW THz spectrometer

The design of our optoelectronic FMCW THz spectrometer is sketched in Fig. 1. We provide a detailed description in the “Methods” section. In order to analyze the performance of the optoelectronic FMCW THz spectrometer, we use a standard transmission setup with two parabolic mirrors, which guide the emitted THz wave to the receiver (see Fig. 1). The THz path length is 40 cm long and the entire setup operates in ambient air. Unless stated otherwise, the tuning bandwidth of the swept laser is 4.7 THz, the frequency step size is 1 GHz and the measurement rate is 68 spectra per second.

**Fig. 4:**
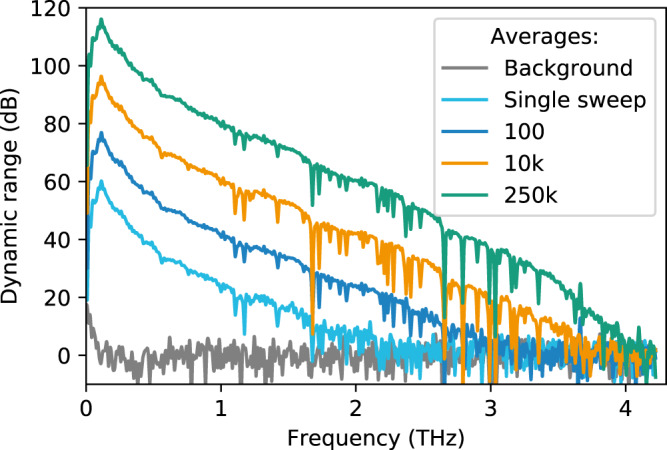
Terahertz spectra recorded with the optoelectronic FMCW THz spectrometer. Spectral sweeps were captured at a repetition rate of 68 Hz and at frequency steps of 1 GHz. The plot shows the amplitude dynamic range for different numbers of averaged spectra, which are normalized to their respective noise level (indicated by gray line). The dips in the spectrum are absorption lines of the atmospheric water vapor.

The DR of THz spectra recorded with the optoelectronic FMCW THz spectrometer for different numbers of averages is shown in Fig. 4. The DR is calculated via

16 $${\mathrm{DR}}\left( f \right)\left[ {{\mathrm{dB}}} \right] = 20\log _{10}\left( {\frac{{\left| {{\mathrm{signal}}\left( f \right) - {\mathrm{background}}\left( f \right)} \right|}}{{{\mathrm{noise}}\,{\mathrm{level}}}}} \right)$$

Here, the complex valued signal(*f*) is a result of the software lock-in procedure. The noise level is calculated from the averaged spectral amplitudes at frequencies above 4 THz. In this frequency range, the signal does not contain any measureable THz components, so we consider it the noise level. The background(*f*) is the signal measured without any bias supplied to the THz emitter. By subtracting the background, any static offset signal is removed. Since emitter and receiver operate beyond their 3 dB cutoff frequency, the DR decreases towards higher THz frequencies^23^. Figure 4 clearly shows an enhancement of both DR and effective bandwidth with a higher number of averages. By effective bandwidth, we denote the highest frequency with a signal well above the noise level. In a single sweep, the complete spectrum is acquired in 17 ms only, and the effective bandwidth and peak DR amount to 2 THz and 60 dB, respectively. The bandwidth extends to almost 3 THz and the peak DR increases by 20 dB when 100 spectra are averaged (1.7 s measurement time). This 10 dB rise of the DR for a factor of 10 in the number of averages is a universal law as long as random noise dominates the signal. This law is derived in the “Methods” section.

**Fig. 5:**
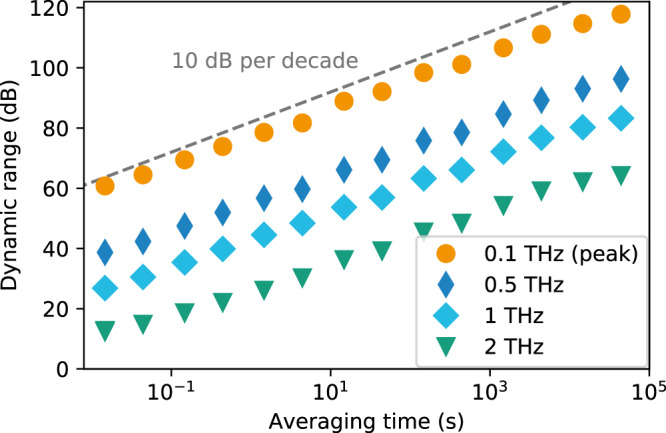
Dynamic range as a function of averaging time for selected frequencies within the THz spectrum. Circles, diamonds, squares, and triangles correspond to frequencies of 0.1 (spectral maximum), 0.5, 1, and 2 THz. The dashed gray line indicates an increase of 10 dB per decade, as predicted by Eq. (20).

In order to verify this behavior for the optoelectronic FMCW THz setup, we evaluated the DR as a function of the measurement time for different frequencies of the THz spectrum (Fig. 5). Circles, diamonds, squares, and triangles represent frequencies of 0.1 (the spectral maximum), 0.5, 1, and 2 THz, respectively. As a guide to the eye, the dashed line shows the expected DR increase of 10 dB per decade, which is fulfilled for all four frequencies up to an integration time of 5000 s (83 min). The averaging is performed on the complex data of amplitude and phase, and consequently, any instabilities in the phase would manifest themselves in strong deviations from the predicted increase (see “Methods” section). Since this is not the case, we conclude that no significant phase drifts, neither in the spectrometer nor in the experimental setup, occur over this period. Small deviation from the 10-dB-per-decade increase are observed for long integration times though. We surmise that these deviations originate from long-term drifts in the THz setup as well as temperature effects that influence the refractive index of the optical fibers. Still, the results shows that our measurement scheme enables very stable measurements with high DR. In particular, the peak DR of 117 dB is the highest value reported for any optoelectronic THz system thus far.

## Discussion

In the previous section, we demonstrated that the optoelectronic FMCW THz system can be used for broadband terahertz spectroscopy with high DR. Currently, one of the most promising industrial applications of terahertz spectroscopy is thickness gauging of thin dielectric layers. As mentioned in the introduction, layer thickness measurements are as yet performed with pulsed THz TDS systems. The main reason, besides the fact that only THz TDS systems are commercially available for this application, is their high bandwidth of up to 6.5 THz and the comparably fast measurement speed up to a few kHz^7,15^. Unlike previous continuous-wave THz concepts, our optoelectronic FMCW THz spectrometer offers a performance comparable to THz TDS systems, i.e. a measurement rate of 68 Hz at a bandwidth of 4 THz, and a measurement rate as high as 200 Hz at a bandwidth of 1.5 THz.

In this section, we apply the optoelectronic FMCW THz system to thickness measurements of multilayer and single-layer dielectrics, and demonstrate an accuracy better than 2 µm. We investigate three different samples: First, we determine the transfer function of a 380-µm-thick wafer made of high-resistivity float-zone (HRFZ) silicon (Si). We demonstrate that even for thickness measurements, the usable bandwidth of the system remains 3 THz with only 15 s of averaging. Second, we measure the thickness of a single-layer dielectric PET foil with a nominal thicknesses of 23 µm. Finally, we determine the individual thicknesses of a triple-layer sample consisting of a ceramic substrate with a nominal thickness of 640 µm, which is coated with green spray paint with a nominal thickness of 40–80 µm (see below). All measurements are conducted in reflection geometry and with dry-air purging. Details on the reflection head and the measurement setup are presented in the “Methods” section.

**Fig. 6:**
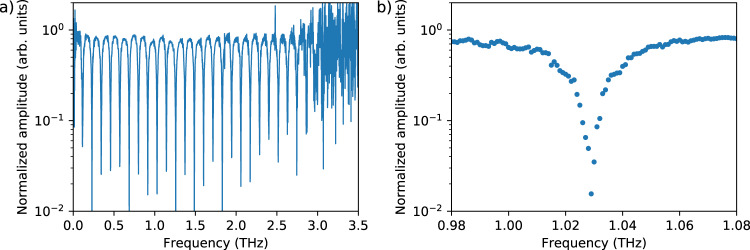
Absolute value of the transfer function of a HRFZ-Si wafer, recorded in reflection geometry with a focused THz beam. The acquisition of 1000 averages leads to a total measurement time of 15 s. The THz path was purged with nitrogen gas to avoid water vapor absorption. The full THz spectrum (**a**) and a magnification of a single Fabry–Pérot dip around 1.03 THz (**b**) are shown.

Figure 6 shows the absolute value of the transfer function of a HRFZ-Si wafer acquired in less than 15 s. We chose silicon because its transfer function is spectrally flat over the entire spectral range. The transfer function exhibits Fabry–Pérot fringes, which result from standing waves inside the wafer. Their free spectral range corresponds to the thickness of the wafer. The Fabry–Pérot fringes are well resolved and their maxima remain flat up to 3 THz, which corresponds to the accessible bandwidth under these operating conditions, which proves that the full bandwidth of the system is available for spectroscopic measurements. The right panel of Fig. 6 shows a magnification of a single Fabry–Pérot dip at 1.03 THz. Note that the frequency resolution of 1 GHz clearly reveals the shape and depth of the dip. By contrast, a pulsed THz system with a typical frequency resolution of 10 GHz would not be able to resolve these narrow dips as well.

**Fig. 7:**
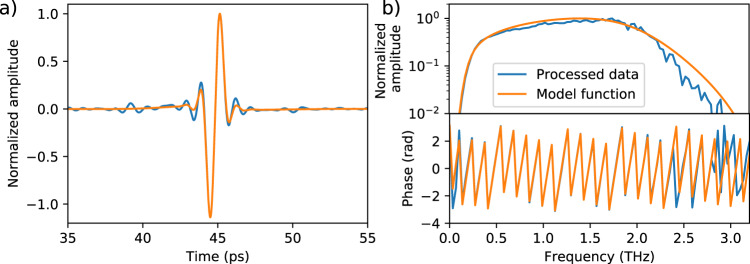
Reflection measurement data and model of a 23 µm PET foil. **a** Time-domain representation of the THz signal reflected from a single-layer PET foil with a nominal thickness of 23 µm (blue) and corresponding model function (orange) used for thickness determination. Details on the model function can be found in the “Methods” section. **b** Spectral amplitude and phase of the measured data and model function. For these measurements, individual spectra were averaged ten times, which corresponds to an acquisition time of less than 0.2 s.

Next, we present thickness measurements on dielectric samples. Prerequisites for accurate and reproducible thickness measurements are a broad spectral bandwidth, high DR, and excellent phase stability. In the first measurement, we evaluate the reflection from a single-layer PET foil with a nominal thickness of 23 µm. Details of the thickness determination are provided in the “Methods” section. Our analysis assumes a frequency-independent refractive index of 1.75 for PET^34^. Figure 7a shows the quasi time-of-flight signal, i.e. the inverse Fourier transform of the complex spectrum, of the PET foil (blue) and the model function (orange) used for thickness determination. Note that the reflections from the front and backside of the thin foil cannot be distinguished as they fully overlap. However, the frequency-dependent signal shown in Fig. 7b reveals that both data and model function feature a similar spectral shape in amplitude and phase. Accordingly, we can determine the sample thickness from the data even if the individual peaks cannot be distinguished in the time-of-flight domain. By fitting the model function to the data, we obtain a thickness of 26 ± 0.3 µm. The uncertainty represents the standard deviation of 50 subsequent measurements of the same sample. This uncertainty of 0.3 µm corresponds to only 1.2 % of the total sample thickness, which underlines the capability of the optoelectronic FMCW THz spectrometer to measure thin dielectric layers with high accuracy. The discrepancy between the measured value and the nominal thickness is attributed to the inhomogeneity of the sample.

**Fig. 8:**
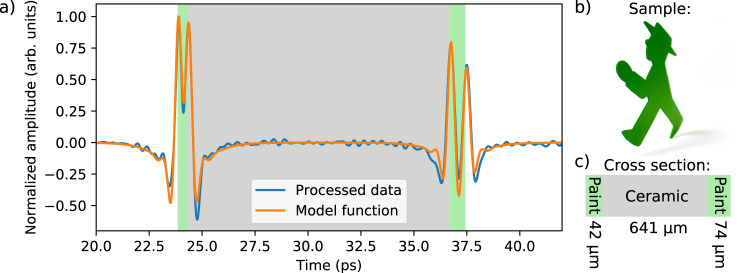
Reflection measurement data and model of a three-layer sample. **a** Processed time-of-flight signal (blue) and model function (orange) of a three-layer sample. The tree layers are indicated by the areas shaded in green and gray. **b** The samples outer shape resembles a traditional Berlin traffic light figure. **c** The three-layer samples inner structure: green paint on the front side, a ceramic substrate, and another green paint layer on the backside. Note that the model function reproduces the individual peaks of the data trace accurately. A sketch of the sample and its cross-section showing the layer stack are depicted on the right.

Following the same procedure, we measured the individual thicknesses of a multilayer sample consisting of a ceramic substrate with green spray paint on both sides. The time-of-flight signal (blue) and the model function (orange) are shown in Fig. 8. The highlighted areas correspond to the green paint layers on the front and backside of the ceramic substrate. The model function reproduces the individual peaks accurately. The fit of the model function yields the following thickness values: (42.5 ± 0.8) µm for the paint layer on the front side, (641.3 ± 0.6) µm for the ceramic substrate, and (74.4 ± 1.3) µm for the thickness of the spray paint on the backside. Note that for all layers, the uncertainty remains well below 2%. This proves that the FMCW THz concept is a promising alternative to THz TDS for industrial NDT, and in particular, for contact-free layer thickness measurements. Due to the simplicity of its architecture and its high measurement speed, the optoelectronic FMCW THz scheme has strong potential to replace complex THz TDS systems in this field of application.

In conclusion, we demonstrated that S-DSH with a rapidly tuned semiconductor laser lends itself to phase-sensitive optoelectronic FMCW THz spectroscopy. Compared to present-day terahertz spectrometers, this measurement scheme is significantly less complex, since it eliminates the need for active phase modulation. Thus, optical phase modulators with high insertion loss, bulky fiber stretchers, or slow optical delay lines are no longer required. The optoelectronic FMCW THz system only consists of two semiconductor lasers, an erbium-doped amplifier, a THz emitter and receiver pair, and a data acquisition unit. It does not even require strictly continuous tuning, so that even telecom lasers with deterministic mode jumps in their tuning range may be used. This very simple system architecture, in conjunction with the fast tuning of the swept laser, enables cw THz measurements with high speed and long-term stability. The main advantage of the optoelectronic FMCW approach compared to strictly electronic FMCW systems is the enormous bandwidth of 4 THz, which leads to higher spatial resolution at comparable measurement rates. We demonstrated measurement rates of 200 Hz for THz spectra with 1.5 THz bandwidth. To the best of our knowledge, the DR of 117 dB and the bandwidth of 4 THz present the highest values reported for continuous-wave THz systems. Furthermore, the spectrometer proved well suited for non-contact thickness measurements of multilayer dielectric samples, an application field that has commonly been addressed with THz TDS techniques. Due to the high complexity of THz TDS systems, however, they have failed to reach widespread industrial acceptance. With our distinctly simpler optoelectronic FMCW THz system, we were still able to measure the thickness of a 23 µm thin PET foil with an accuracy of 0.3 µm. A multilayer sample, consisting of a ceramic substrate with 40–80 µm spray paint on both sides, could also be measured with high accuracy. The standard deviation of consecutive measurements remained well below 2% for all three layers. Hence, the utilization of the S-DSH scheme in cw THz spectroscopy is an important step to simplify the technology as a whole, which directly translates into higher reliability, lower assembly effort, and better cost scaling. This demonstration is just the starting point for further miniaturization and compactification of cw THz spectrometers. As the main components of the system can be fabricated in semiconductor technology platforms, photonic and electronic co-integration will pave the way towards miniaturized cw THz systems. In combination with on-chip optical phased arrays, even handheld THz scanners become conceivable. Hence, this measurement scheme may open the door to a variety of additional applications in sensing, imaging, and broadband spectroscopy.

## Methods

### Swept laser

The Finisar® WaveSource™ is the swept laser source used throughout this work. It is based on a modulated-grating Y-branch laser (MG-Y)^35^. Current injection into two multi-peak reflectors is used for fast and wide frequency tuning, while the Vernier effect allows controlling the lasing frequency. Thus, the laser sweeps fast and in a fully deterministic manner across the entire c-band. An internal wavelength locker ensures an absolute frequency accuracy of typically 300 MHz. Predefined sweep modes need to be calibrated, and are subsequently recalled from a look-up table to ensure deterministic operation. The frequency reproducibility between different sweeps is around 50 MHz, and the variation of the output power with frequency remains below 1 dB. The experiments were performed with a step size of 1 GHz and two different tuning ranges: either 1.5 THz or the full c-band. In both scenarios, the dwell time was *τ*_s_ = 2 µs, and the effective tuning speed $\nu_{\mathrm{S}}$ was 500 THz/s. Well-defined mode jumps occur during tuning, which are indicated by the specific trigger scheme implemented in the laser. In a previous publication, we showed that these mode jumps do not influence the THz measurements if they are cut out of the data stream^25^. The total duty cycle of the laser is around 70–80% including all mode transitions and the reset time between subsequent sweeps.

### MZI for swept laser characterization

The MZI is essentially the S-DSH setup and serves as a simple test platform for the sweeping laser, without having to account for the influence of the THz-specific components. The setup is depicted in Fig. 3c) based on telecom fiber optics. Delay time *T* and therefore also the detector frequency *f*_0_ differ slightly from the FMCW THz setup; therefore, the values specific to the MZI are indicated by the superscript MZI. The interferometer features polarization-maintaining optical fibers and in-fiber 3 dB couplers. The interference signal is recorded by a photodiode (PD, Thorlabs DET 10C) connected to an electrical spectrum analyzer. A fiber length difference of 20.6 cm between both arms of the interferometer introduces a static time delay of *T*^MZI^ = 1 ns. With 500 THz/s frequency tuning speed of the laser, the central intermediate frequency measured with the PD amounts to $f_0^{{\mathrm{MZI}}} = 498{\,}{\mathrm{kHz}}$.

### Technical details of the optoelectronic FMCW THz setup

The optoelectronic FMCW THz setup, as used throughout the paper, is shown in Fig. 1. All of the optical components are standard building blocks designed for telecom applications, and operate in the c-band (1526.9–1568.5 nm). The setup is based on fiber optics for robust and alignment-free handling of the components. The swept laser is described in the “Methods” section. The fixed-frequency laser, a CoBrite DX1 from ID Photonics, emits at 1565 nm and has a linewidth below 100 kHz. A polarization-maintaining (PM) 3 dB fiber-coupler combines the beams of both lasers. The resulting beat note is amplified to about 70 mW with an EDFA (GOA-SP184 from BKtel photonics) and split by a second 3 dB coupler into two arms with an optical power of 32 mW each. The optical signals of the two arms are guided to the fiber-coupled THz emitter and receiver, respectively. The THz emitter and receiver are based on a commercially available waveguide-integrated PIN diode and a photomixing receiver, respectively, from Toptica Photonics AG^36^. The THz beam path consists of two 90° off-axis parabolic mirrors. The receiver current is amplified by a Femto DLPCA-200 transimpedance amplifier with a gain of 10^5^ V/A. The receiver current is low-pass filtered (3 dB frequency of 500 kHz) and digitized with 12 bit and 5 MS/s. After clearing the signal of the deterministic mode jumps of the swept laser, it is analyzed with a quadrature software lock-in: the signal is band-pass filtered (414–511 kHz) and multiplied by the complex function exp(*i*2*πf*_LI_*t*), where the lock-in frequency *f*_LI_ is close to the effective intermediate frequency *f*_0_ = 455 kHz. The resulting complex signal is processed and summed up for each time interval of *τ*_s_, which yields amplitude and phase as a function of frequency.

### DR as a function of averaging

Consider a complex signal *S* = *A*exp *jϕ*, which is recorded *N* times. Assuming that the signal amplitude *A* does not change significantly within the recording period, the signal amplitude after *N* averages can be written as

17 $$A_N = \left| {S_N} \right| = \left| {\mathop {\sum}\limits_{i = 1}^N {S_i} } \right| = \left| {\mathop {\sum}\limits_{i = 1}^N {A_i{\mathrm{e}}^{j\phi _i}} } \right| \approx E\left[ {A_1} \right]\left| {\mathop {\sum}\limits_{i = 1}^N {{\mathrm{e}}^{j\phi _i}} } \right| = E\left[ {A_1} \right]\eta N.$$

Here, *S*_*i*_ is the *i*th recording of the signal, E[*A*_1_] is the single-shot expected value and 0 < *η* < 1 is the contrast reduction due to random fluctuations of the phase angle. The central limit theorem dictates that *η* is unity for a perfectly aligned phase angle *ϕ*_*i*_, and 1/√*N* in case of random phase and large *N*. Essentially, *η* is a measure of the signal reduction caused by phase drifts within the number of averages. Noise contributions *s*_*i*_ that add the signal recording *S*_*i*_ can be characterized by a random amplitude distributed around the single-shot expectation value E[*a*_1_] and random phase *ξ* with *η* = 1/√*N*

18 $$a_N = \left| {s_N} \right| = \left| {\mathop {\sum}\limits_{i = 1}^N {S_i} } \right| = \left| {\mathop {\sum}\limits_{i = 1}^N {a_ie^{j\xi _i}} } \right| \approx E\left[ {a_1} \right]\sqrt N .$$

Hence, the DR, i.e. the ratio of signal to noise, after *N* averages reads

19 $${\mathrm{{DR}}}_N = \frac{{A_N}}{{a_N}} \approx \eta \sqrt N \frac{{E\left[ {A_1} \right]}}{{E\left[ {a_1} \right]}}.$$

In decibel, the DR_*N*_ is

20 $${\mathrm{{DR}}}_N\left[ {{\mathrm{dB}}} \right] = 20\log _{10}\left( {\frac{{A_N}}{{a_N}}} \right) \equiv {\mathrm{{DR}}}_1\left[ {{\mathrm{dB}}} \right] + 10\log _{10}N + 20\log _{10}\eta .$$

Equation (20) shows that the DR increases maximally 10 dB if the number of averages *N* increases by a factor of 10. A dashed gray line in Fig. 5 highlights this 10 dB increase for the spectra averaged with the FMCW THz system. From Eq. (20), it is also evident that two factors can reduce this 10 dB rise: First, the noise may not be completely random and second, a phase drift within the averaging time leads to *η* < 1.

### Thickness measurements

All thickness measurements are performed in reflection geometry with a mirror-based reflection head. This is realized by modifying the THz path from the sketch in Fig. 1. The THz beam propagates as follows: A 90° off-axis parabolic mirror with 2 inch focal length collimates the THz beam of the emitter, and another 90° off-axis parabolic mirror with 4 inch focal length focuses the beam on the sample surface under an angle of 8°. The same configuration of mirrors guides the reflected THz beam to the photoconductive detector. In order to avoid THz absorption from water vapor, the volume between sensor and sample under test is purged with nitrogen gas. For thickness determination, the following procedure is used: First, we use an aluminum reflector to record a reference measurement. Thereby, the frequency for quadrature lock-in detection *f*_LI_ is set to be close to *f*_0_, which is indicated by a mostly flat spectral phase. Then, we replace the reference aluminum reflector with the sample and record its THz reflection spectrum. In the raw signal, each reflecting interface appears as single frequency. After the software lock-in, the amplitude and phase spectra show the complex structure of Fabry–Pérot interference, combined with the typical dynamic-range roll-off towards higher frequencies. For both reference and the sample, we average ten individual spectra, which corresponds to an acquisition time of less than 0.2 s. Next, the complex transfer function of the sample is calculated by dividing the sample spectra by the reference data. By an inverse, real-valued Fourier transformation (iFFT), the complex transfer function is converted into the time-of-flight domain, where individual peaks indicate material interfaces^37^. Finite impulse response (FIR) Bessel filters are employed to remove high-frequency artefacts as well as the DC background, both of which originate from the division of the two spectra and the iFFT. In order to extract the physical thickness in a deterministic and reproducible way, we fit a very simple model to the time-of-flight data. Each interface is represented by an infinitesimally narrow peak in the time-of-flight domain, and subsequently filtered with exactly the same FIR filters as the measured signal. This signal template is then fitted to the data by varying the amplitudes and positions of the peaks. Afterwards, the physical thickness can be extracted from the peak-to-peak distance of the template, by scaling with the refractive index of the material. This simple model fitting procedure requires prior knowledge about the sample, in particular its number of layers and their refractive index. It does not account for material absorption or any frequency dependence of the refractive index. We chose this simple procedure in order to demonstrate that the quality of our data is so high that even simple thickness algorithms achieve good results. We are confident that more advanced evaluation techniques will produce even better results.

## Data Availability

The data that support the findings of this study are available from the authors on reasonable request.

## Code availability

All codes written for and used in this study are available from the corresponding authors upon reasonable request.
